# Exploring the experiences, priorities and preferences of people living with Parkinson’s on exercise and physical activity promotion in the UK

**DOI:** 10.1371/journal.pone.0304223

**Published:** 2024-06-12

**Authors:** Ledia Agley, Peter Hartley, Louise Lafortune

**Affiliations:** 1 Department of Public Health and Primary Care, University of Cambridge, Cambridge, United Kingdom; 2 Department of Physiotherapy, Cambridge University Hospital NHS Foundation Trust, Cambridge, United Kingdom; 3 Cambridge Public Health, Interdisciplinary Research Centre, University of Cambridge, Cambridge, United Kingdom; University of North Texas Health Science Center, UNITED STATES

## Abstract

**Background:**

People with Parkinson’s (PwP) want access to timely, relevant, and specific exercise and physical activity (PA) information to enable them to manage their symptoms and maintain wellbeing and quality of life. Research that promotes exercise in this population group is limited. Little is also known about the clinical practice around PA promotion in this population, especially around the time of diagnosis.

**Objective:**

To explore the experiences, preferences, and priorities of PwP around exercise and PA promotion and assess their knowledge on these topics.

**Methods:**

A cross-sectional online survey for PwP in the United Kingdom was conducted from July to December 2021.

**Results:**

430 participants started the survey and 405 completed it. Participants had a mean age of 65.1 (±9.2) and had been living with Parkinson’s for a varying time (up to 2 years = 38%, up to 6 years = 39% and for 7 or more years = 23%). Most participants reported they had not received an education (68%; n = 276) or exercise intervention (54%; n = 217) as part of their routine management by the National Health Service (NHS) since diagnosis and had sought services privately. Knowledge of the overall benefits of exercise was good, however participants lacked specific knowledge on the impact of Parkinson’s Disease (PD) on posture, falls and muscle strength. 90% of participants reported they would participate in an exercise and PA education interventions.

**Conclusions:**

PwP want exercise and PA education interventions that provide knowledge, skills and access to opportunities that enable participation. For the majority, these interventions have not been provided as part of their routine care pathway. To align with the priorities and preferences of PwP, interventions should be offered around the time of diagnosis, include content that is relevant and specific to how exercise and PA can mitigate symptoms of PD and should be delivered in person or online in a group setting.

## Introduction

Evidence for the role of exercise and physical activity (PA) in the management of Parkinson’s Disease (PD) is increasing. This includes evidence in both standardised exercise-based protocols, including aerobic [1, 2] strength [3], and balance training programs [4] as well as in less clinically conventional forms of exercises such as dance [5], Nordic walking [6] and Tai-Chi [7]. The evidence shows positive impact on motor and non-motor impairments, leading to an improved quality of life for people with Parkinson’s (PwP) [8]. The effect of structured exercise on balance, postural instability and gait are particularly encouraging considering both the progressive nature of PD and the limited effect of medication on these symptoms [9]. PwP have listed access to information and opportunities about exercise and PA as one of their top 30 wishes to aid them understand and manage their condition better [10].

In the United Kingdom (UK), the National Institute for Health and Care Excellence (NICE) recommends PA educational intervention to occur around the time of diagnosis in order to promote healthy behaviours [11]. This is supported by research demonstrating that knowledge of the benefits of exercise on PD symptoms and knowledge on the frequency and type of exercises to do are perceived as motivators to exercise by PwP [12–14]. However, little is known of current practice in the UK, or of the knowledge gaps, needs and preferences of PwP relating to exercise and PA education. Gaining this knowledge is seen as a key step in the successful implementation of the NICE guidelines [11], and in fulfilling a ‘top 30 wish’ of PwP to aid them understand and manage their condition better [10].

The primary objectives of this study are therefore:

- to explore the practice around education and information provision for PwP in the UK;
- to identify the gaps in the knowledge level of PwP in the UK around exercise and PA;
- to identify the needs and preferences of PwP in the UK on exercise and PA education provision around the time of diagnosis.

A secondary objective of this survey was to understand and explain the potential relationship between knowledge levels of PwP in relation to exercise and PA, and PA behaviour.

## Methods

### Study design

This was a cross-sectional online survey for people living with Parkinson’s in the UK. The protocol for this study received local Ethics approval by the Cambridge Psychology Research Ethics Committee (PRE.2021.039).

### Sample and recruitment

Convenience sampling methods were utilised for the recruitment. These methods display a number of advantages and disadvantages, however, they are widely used and remain an effective and pragmatic solution in survey design [15]. Participants were recruited via the Parkinson’s UK charity and its digital channels. An advert of the study was also placed in the social media pages (with consent) of charities and associations such as Cure Parkinson’s, Neurology Academy and Parkinson’s Foundation. Further to the online dissemination, local PD research clinics sent out an advert for the study via their postal newsletter, asking interested participants to call the PI should they be interested in participating in the study. Local support groups were also approached to recruit older adults with PD known to them, who might have difficulty accessing the internet.

The survey was written in English and therefore was limited to people speaking and understanding the English language. There were no exclusion criteria based on years since diagnosis. Participants over the age of 18, with a diagnosis of Parkinson’s, living in the UK were invited to take part in the survey. Those interested in the study followed a link to the online survey and landed on the participant information sheet followed by the consent form page. The consent form included statements asking people to confirm they were over 18 years old, had a PD diagnosis, had read the participant information sheet, and understood all the information provided, and knew who to contact should they need to. Only those who consented by clicking “yes” for all statements could access the survey questions. If they clicked “no”, they were thanked for their time and provided with the Principal Investigator contact details should they wanted more information.

The survey was live from the 14th of July 2021 and received the last response on the 16^th^ of December 2021 when the survey was closed. No further responses were received past that date online or on a paper-form.

### Survey

The survey for this study was designed according to the survey design guidelines of Kelley et al. [16]. The content of the survey was informed by findings from a systematic review of the literature on education interventions around exercise and PA for PwP [17], as well as previous survey exploring beliefs, preferences of PwP regarding exercise and PA [18]. Input was sought from a Patient and Public Involvement (PPI) group, which included PwP, academics and clinicians who had experience and expertise in PD.

The survey comprised four parts: participant demographics; questions exploring practice around exercise and PA promotion during routine clinic appointments; questions exploring knowledge of exercise and PA; and questions on preferences and priorities around exercise and PA education interventions including on the content and mode of delivery of PA and exercise education for people newly diagnosed with Parkinson’s (S1 Table).

The following demographic variables were collected: age, sex, ethnicity, time since diagnosis, education level, number of chronic conditions, current ability to perform activities of daily living (ADL), minutes of moderate to vigorous intensity physical activity (MVPA) per week. All variables were self-reported by participants. Chronic conditions were entered in a free text box and tallied by the research team.

Time since diagnosis was categorised as 0–6 months, 7–11 months, 1–2 years, 3–4 years, 5–6 years, 7–8 years, 9–10 years and over 10 years. Education levels were categorised using the UK governments classification system [19] and grouped as follows: up to a Level 3 qualification (A-level or equivalent); or to a Level 4 qualification or above. A-levels are typically completed at aged 18 in the UK. Lever 4 includes university undergraduate and postgraduate degrees.

Participants rated their ability to perform ADL as one of five categories: no effect of PD on ADL performance; independent but slower with ADL; require assistance with more demanding ADL tasks; require assistance with most ADL tasks; require assistance with all ADL tasks.

Participants were asked to estimate the amount of self-reported minutes of moderate intensity activity they performed on average per week (up to a maximum of 200min). Moderate intensity was defined as activities that cause increase in breathing, for example brisk walking, pushing a lawnmower or cycling uphill.

To explore knowledge regarding exercise and physical activity in PD—a key dimension of physical literacy [20], a nine-question Knowledge Exercise and Physical Activity (KEPA) PD questionnaire was designed (S1 Table) based on the content of exercise education interventions identified in a systematic review of the literature [17]—each statement conveyed evidence from existing literature. A score of one was given for correct answers and no points were given for incorrect answers or where the participant answered ‘not sure’. Participants scores were calculated by summing the number of correct questions (ranging from 0 to 9, with higher scores indicating a higher level of knowledge).

The survey was pilot tested by 15 PwP independent of the participants of the study to assess content and face validity as well as language accessibility. Upon integrating the feedback from pilot testing, the survey was administered online via Qualtrics (https://www.qualtrics.com, Qualtrics, Provo, UT), an online survey tool approved by the University of Cambridge for academic research. Paper-form surveys and free return envelopes were available for participants with no access to internet and those who preferred a paper version.

### Analysis

Data were analysed with R software [21]. Descriptive statistics were performed and presented as mean with standard deviation (± SD) and median with interquartile range (IQR) or count with percentage (%) to report the demographic and clinical characteristics of participants. Likert type questions were treated as ordinal data and responses were presented as percentages to describe variability in current practice around education provision, and preferences around exercise and PA education interventions. The Likert questions for examining the provision of exercise and PA education or information since diagnosis of PD were also grouped by time from PD diagnosis (0–2 years, 3–6 years, 7–10+ years), to look for differences in exercise and PA promotion over time.

To explore relationship between exercise and PA knowledge and self-reported MVPA values a median regression model was produced using the quantreg package [22]. This was preferred to a linear model as 30% of respondents had entered the maximum 200 minutes of MVPA per week that the survey allowed. The KEPA PD score was the independent variable; the co-variates were sex, age, highest educational level achieved (up to a Level 3 qualification, Level 4 or higher), and ADL score (1–5 as described above). In the literature, some of these covariates have been previously suggested as possible determinants of PA and exercise behaviour in the PD population [23–25].

A pseudo-R2 statistic was calculated using methods described by Koenker & Machado [26].

## Results

### Participant demographics

Of the 430 people who took part in the survey, 405 completed it in full, resulting in a 94% completion rate. Of those who completed, 185 respondents were female and 218 were male. Almost half of the respondents (47%) had been living with Parkinson’s for up to 4 years and were independent with everyday activities (82%). All respondents’ characteristics are shown in Table 1.

**Table 1:** Participant demographic and clinical characteristics.

Variables		All (n = 430)	Completed survey in full (n = 405)	Started but did not finish survey (n = 25)
**Age** (mean, SD)		65.1 (±9.2)	65.1 (±9.2)	64.0 (±10.0)
**Sex**				
	Female	201 (46.7%)	185 (45.7%)	16 (64.0%)
	Male	227 (52.8%)	218 (53.8%)	9 (36.0%)
	Prefer not to say	2 (0.5%)	2 (0.5%)	0 (0.0%)
**Self-reported minutes of moderate activity/week**		138.3(±63.0)	138.8(±63.1)	118.4(±57.9)
**Ethnicity**				
	White	417 (97.0%)	394 (97.3%)	23 (92.0%)
	Asian	7 (1.6%)	5 (1.2%)	2 (8.0%)
	Black	1 (0.2%)	1 (0.2%)	0 (0.0%)
	Hispanic	1 (0.2%)	1 (0.2%)	0 (0.0%)
	Mixed	1 (0.2%)	1 (0.2%)	0 (0.0%)
	Other	1 (0.2%)	1 (0.2%)	0 (0.0%)
	Prefer not to say	2 (0.5%)	2 (0.5%)	0 (0.0%)
**Education level**				
	Up to a Level 3 qualification (A-level or equivalent)	135 (31.4%)	128 (31.6%)	7 (28.0%)
	Education to a Level 4 qualification or above	286 (66.5%)	268 (66.2%)	18 (72.0%)
	Prefer not to say	9 (2.1%)	9 (2.2%)	0 (0.0%)
**Time since diagnosis**				
	0–6 months	30 (7.0%)	30 (7.4%)	0 (0.0%)
	7–11 months	29 (6.7%)	27 (6.7%)	2 (8.0%)
	1–2years	107 (24.9%)	97 (24.0%)	10 (40.0%)
	3–4 years	96 (22.3%)	93 (23.0%)	3 (12.0%)
	5–6 years	71 (16.5%)	66 (16.3%)	5 (20.0%)
	7–8 years	32 (7.4%)	31 (7.7%)	1 (4.0%)
	9–10 years	18 (4.2%)	17 (4.2%)	1 (4.0%)
	Over 10 years	47 (10.9%)	44 (10.9%)	3 (12.0%)
**Self-reported ability on activities of daily living**				
	0 = No effect of PD in ADL performance	63 (14.7%)	61 (15.1%)	2 (8.0%)
	1 = Independent but slower with ADL	288 (67.0%)	270 (66.7%)	18 (72.0%)
	2 = Requires assistance with more demanding ADL tasks	74 (17.2%)	69 (17.0%)	5 (20.0%)
	3 = Requires assistance with most ADL tasks.	3 (0.7%)	3 (0.7%)	0 (0.0%)
	4 = Requires assistance with all ADL tasks	2 (0.5%)	2 (0.5%)	0 (0.0%)
**Chronic conditions**				
	1	285 (66.3%)	267 (65.9%)	18 (72.0%)
	2	83 (19.3%)	80 (19.8%)	3 (12.0%)
	3	40 (9.3%)	37 (9.1%)	3 (12.0%)
	4	7 (1.6%)	7 (1.7%)	0 (0.0%)
	5	1 (0.2%)	1 (0.2%)	0 (0.0%)
	6	1 (0.2%)	1 (0.2%)	0 (0.0%)

### Education/information provision practice for people with PD

Participants were asked to report whether they had received any PD specific education or exercise intervention since diagnosis. Most respondents reported not having taken part in an education intervention (n = 276/430) nor an exercise class (n = 217/430) since their diagnosis. Forty-two were unsure (n = 40 for education and n = 2 for exercise class). Two hundred and thirty participants out of those who did not receive education (n = 230/276), reported they were not offered any education intervention, nine participants reported they were not interested and eight were not aware that these interventions were available. Most participants who reported having received education (n = 114/154) and exercise classes (n = 199/213), had funded these privately (Table 2).

**Table 2:** Reported providers of exercise classes and education programmes attended.

Providers	Education intervention	Exercise intervention
	n = 114	n = 199
**Private company**	44	132
**Not specified**	27	34
**Charity**	15	7
**National Health Service**	12	14
**Research Study**	10	2
**Multiple providers**	3	7
**Not answered**	3	-
**Developed own**	-	5

Survey participants were asked to think back to the time they were first diagnosed and report on the provision of exercise and PA interventions they received. Participants somewhat to strongly disagreed that they had received a satisfactory amount of information on the benefits of exercise in Parkinson’s (59%) and on information about local exercises resources (68%). Barriers previously identified in the literature on exercise participation such as poor understanding of exercise information given (63%) and uncertainty over exercise outcomes (58%), were also echoed by the participants in this study (Table 3).

**Table 3:** Education/information provision on exercise and PA around the time of diagnosis.

*Thinking back to the time you were firstly diagnosed; how strongly do you disagree or agree with the following statements*. *Around the time of diagnosis*:	Strongly Disagree	Somewhat Disagree	Neither agree nor disagree	Somewhat Agree	Strongly Agree
I received a satisfactory amount of information about the benefits of exercise in Parkinson’s.	129 (31)	114 (28)	49 (12)	75 (18)	45 (11)
I was given a satisfactory amount of information about available classes and activity groups near me.	202 (49)	78 (19)	53 (13)	48 (8)	31 (8)
I was given an exercise programme specific to my needs and symptoms.	276 (67)	49 (12)	36 (9)	30 (7)	21 (5)
I understood the exercise information I was given.	95 (23)	32 (8)	172 (42)	66 (16)	47 (11)
I knew which exercises to complete in order to manage my symptoms.	176 (43)	81 (20)	66 (16)	61 (15)	28 (7)
I knew what outcomes to expect from the exercises I was given.	173 (42)	65 (16)	108 (26)	50 (12)	16 (4)
I was confident I had a good level of knowledge about the role of exercise in Parkinson’s.	132 (32)	81(20)	60 (15)	96 (23)	43 (10)
I wanted to know more about the role of exercise and physical activity in Parkinson’s	22 (5)	19 (5)	59 (14)	116 (28)	196 (48)

*This question was answered by 412 participants. Values are shown as n (%)

Exploring differences in education/information provision practice over time according to the years participants had been living with PD reveal that 18% of participants living with PD for more than 7 years reported to have received satisfactory amount of information on the benefits of exercise versus 32% of participants who had living with the condition for two or less years (32%). However, 80% of participants diagnosed in the last two years, somewhat to strongly agreed they wanted to know more about the role of activity and exercise in Parkinson’s, in comparisons to 63% of those who’ve been living with PD for more than 7 years (S1 Fig).

### Identifying existing knowledge gaps around the role exercise and PA in Parkinson’s management

**Fig 1:**
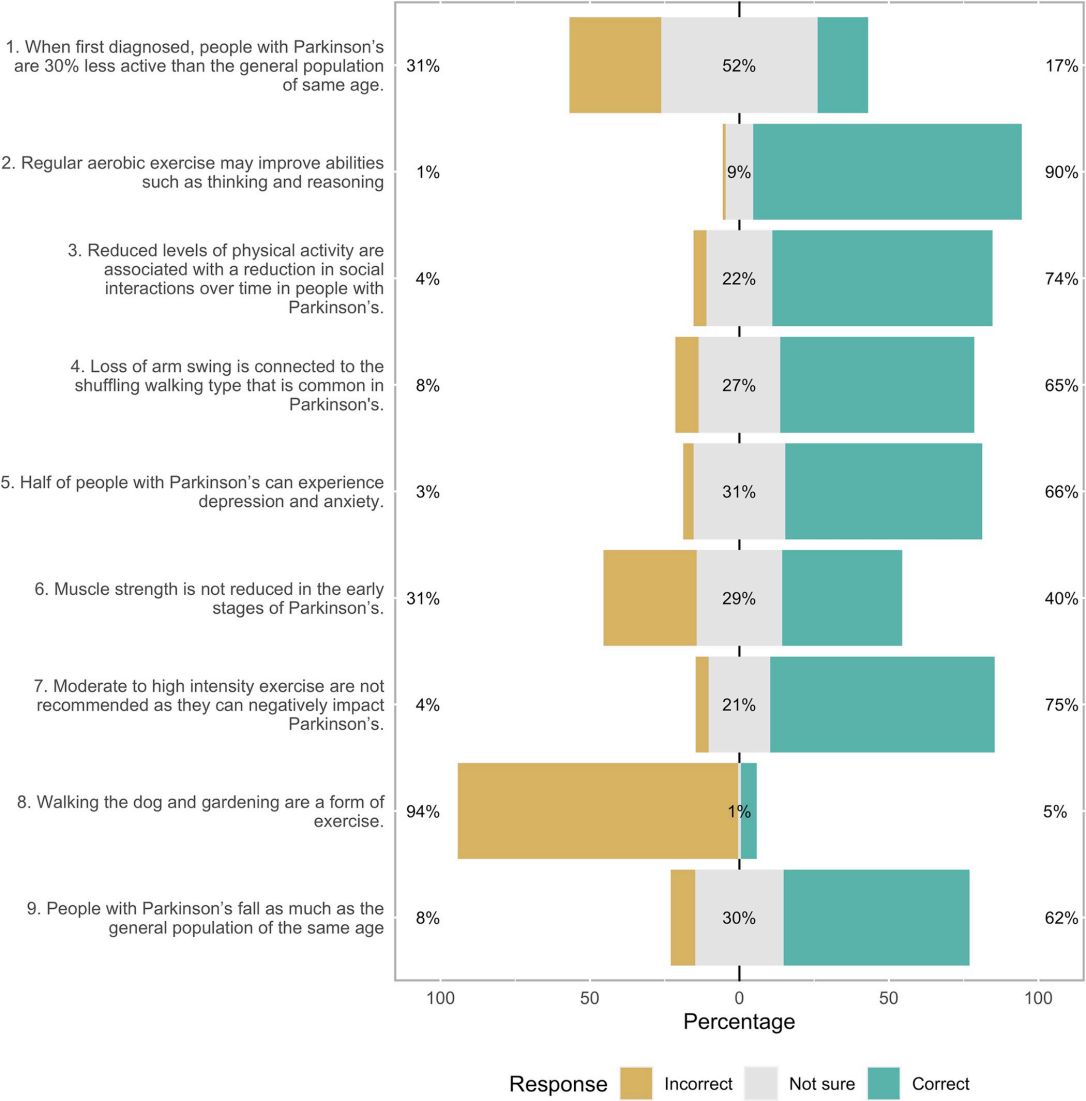
Answers to the knowledge of exercise and physical activity in PD questionnaire.

The KEPA PD questionnaire was completed by 413 participants. No respondents answered all questions correctly: 20 had a 0/9 score either because they answered incorrectly or were unsure of the answer. Most of respondents (65%) had good knowledge on the general benefits of exercise and PA, such as improving cognitive abilities like thinking and reasoning. However, specific knowledge around the impact of PD on muscle strength was low with 40% of participants answering this question correctly. Participants were not able to correctly distinguish between activities that classify as exercise and those that classify as PA, with only 17% correctly identifying walking the dog as a PA (Fig 1).

Correct statements include statement 1, 2, 3, 4, 5,

The results of the median regression model examining the association between age, sex, ADL score, education level, KEPA PD score and self-reported minutes of MVPA per week are presented in Table 4 (pseudo-R^2^ = 0.09). An increase of 5 years in age was estimated to be associated with a reduction of 9.84 minutes (95% CI 8.33 to 11.34) of self-reported MVPA. An increase of 1 level of ADL dependency was associated with a reduction of 22.71 minutes (95% CI 17.11 to 28.3) of self-reported MVPA. KEPA score was not associated with reported MVPA minutes.

**Table 4:** Median regression model of variables associated with self-reported moderate to vigorous physical activity.

Variable	Estimate (minutes)	T statistic	P value	95% CI
**Age**	-1.97	-3.68	< .001	-3.02 to -0.92
**Sex = male**	-3.06	-0.31	0.755	-22.38 to 16.25
**ADL score**	-22.71	-2.94	0.003	-37.89 to -7.53
**Education level = a Level 4 qualification or above**	19.68	1.73	0.084	-2.64 to 41.99
**KEPA score**	-0.32	-0.11	0.913	-6.12 to 5.48

*ADL score: Activities of daily living score out of 5, higher number indicating more difficultly with ADLs

KEPA PD score: Knowledge Exercise and Physical Activity in PD questionnaire, score out of 9, higher number indicating better knowledge.

### Preferences and views on exercise and PA education intervention

**Fig 2:**
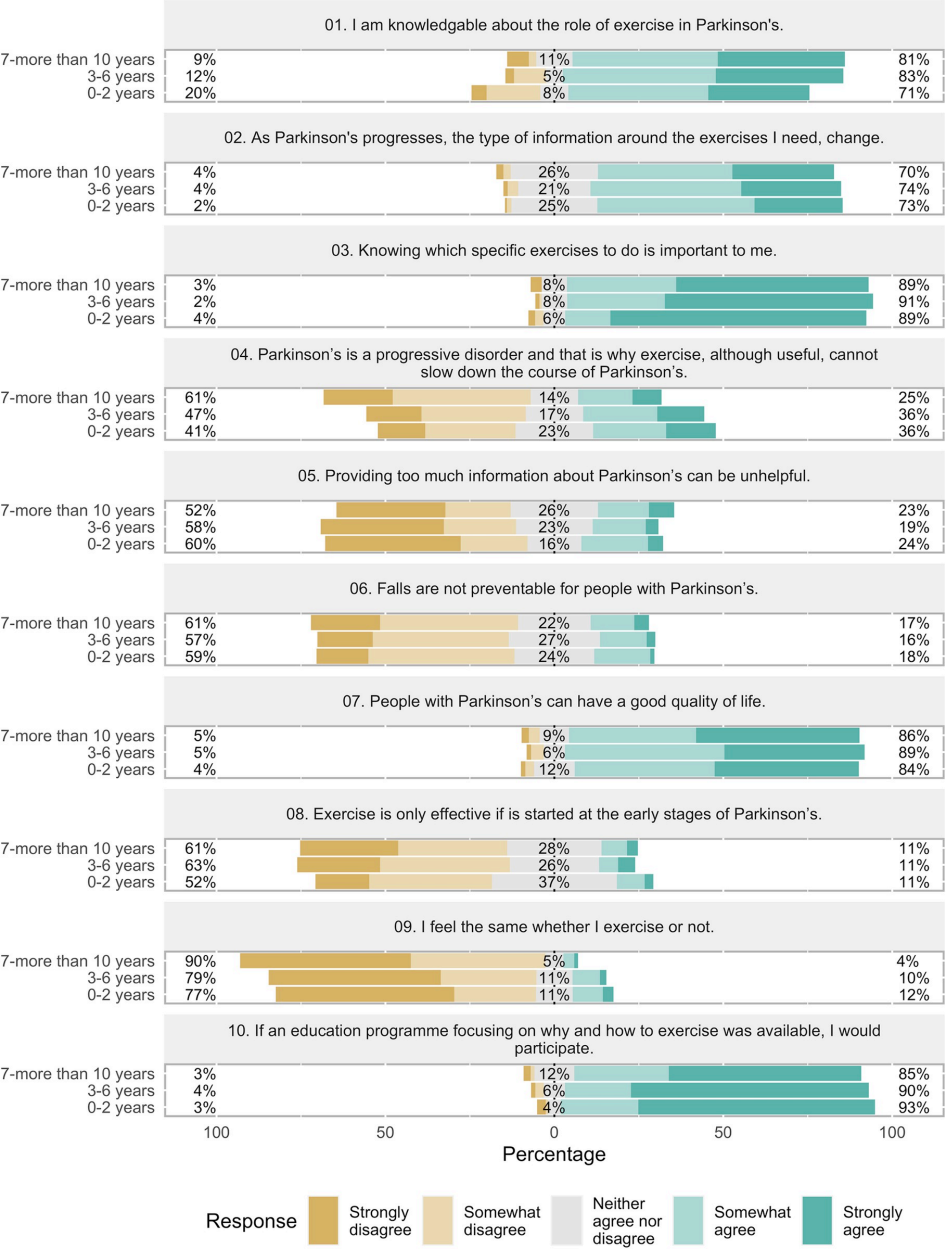
Perceptions and beliefs around the role of exercise and PA according to years living with Parkinson’s.

Likert type statements explored participants current preferences, views on exercise and PA education intervention as well as perceived understanding of their role in PD management. 78% somewhat to strongly agreed that they were knowledgeable about the role of exercise in PD management. Participants had a positive outlook towards the role of exercise and PA in the management of Parkinson’s, with 81% somewhat to strongly disagreeing with the statement that they felt the same whether they exercised or not. Participants somewhat to strongly agreed (93%) they would participate in an education programme that provided information on the importance of exercising and how to exercise with Parkinson’s. The content of education was also important to participants who somewhat to strongly agreed that, as PD progresses, the type of information they require around exercise changes (73%). Receiving information that is relevant and specific to their needs was deemed important by 90% of respondents (S2 Fig). When exploring perceptions around exercise and PA according to the number of years lived with PD, it was noted that a higher percentage of PwP living with the condition for 7 or more years reported being knowledgeable of the role of exercise in the management of PD (81% versus 71% in the 0–2 years group). Furthermore, 61% of PwP living with the condition for 7 or more years perceived that exercise is useful and that it can slow down the course of Parkinson’s, in contrast to 41% of PwP diagnosed in the last 2 years (Fig 2).

Regarding the content of interventions that aimed to improve exercise specific knowledge, participants prioritised topics that focused on evidence-based advice, translation of emerging scientific findings around exercise and PA in the management of Parkinson’s and providing information and exercises relevant to their symptoms. These topics were chosen as the top seven topics to be included in an exercise and PA education intervention for people who are newly diagnosed with PD (Table 5).

**Table 5:** Top seven topics to include in a PA educational intervention.

Topics	Counts (%)
**How exercise impacts motor and nonmotor symptoms**	341 (79)
**The best exercise for my symptoms**	337 (78)
**Current evidence on exercise**	331 (77)
**Pain, stiffness, balance: what is PD what is ageing**	228 (53)
**Evidence of tai chi, dancing, and Nordic walking in PD**	214 (50)
**How to progress my exercise programme**	194 (45)
**Setting meaningful goals for me**	170 (40)
**Information about support groups**	140 (33)
**Falls risks in Parkinson’s**	137 (32)
**How to work with HCP to achieve my goals**	105 (24)
**What is exercise and what physical activity**	89 (20)
**Exploring common barriers to exercise**	75 (17)
**When would I see benefits of exercising**	64 (15)
**What to do if I am not interested in exercises**	38 (8)
**Other**	20 (5)
**Ways to motivate myself exercise more**	4 (1)

Preferences regarding the mode of delivery were also explored. Respondents’ most preferred choice was for in person 1 to 1 interaction with a clinician (46%), followed by online videos (44%), online group discussion (26%) and online reading materials (24%).

**Fig 3:**
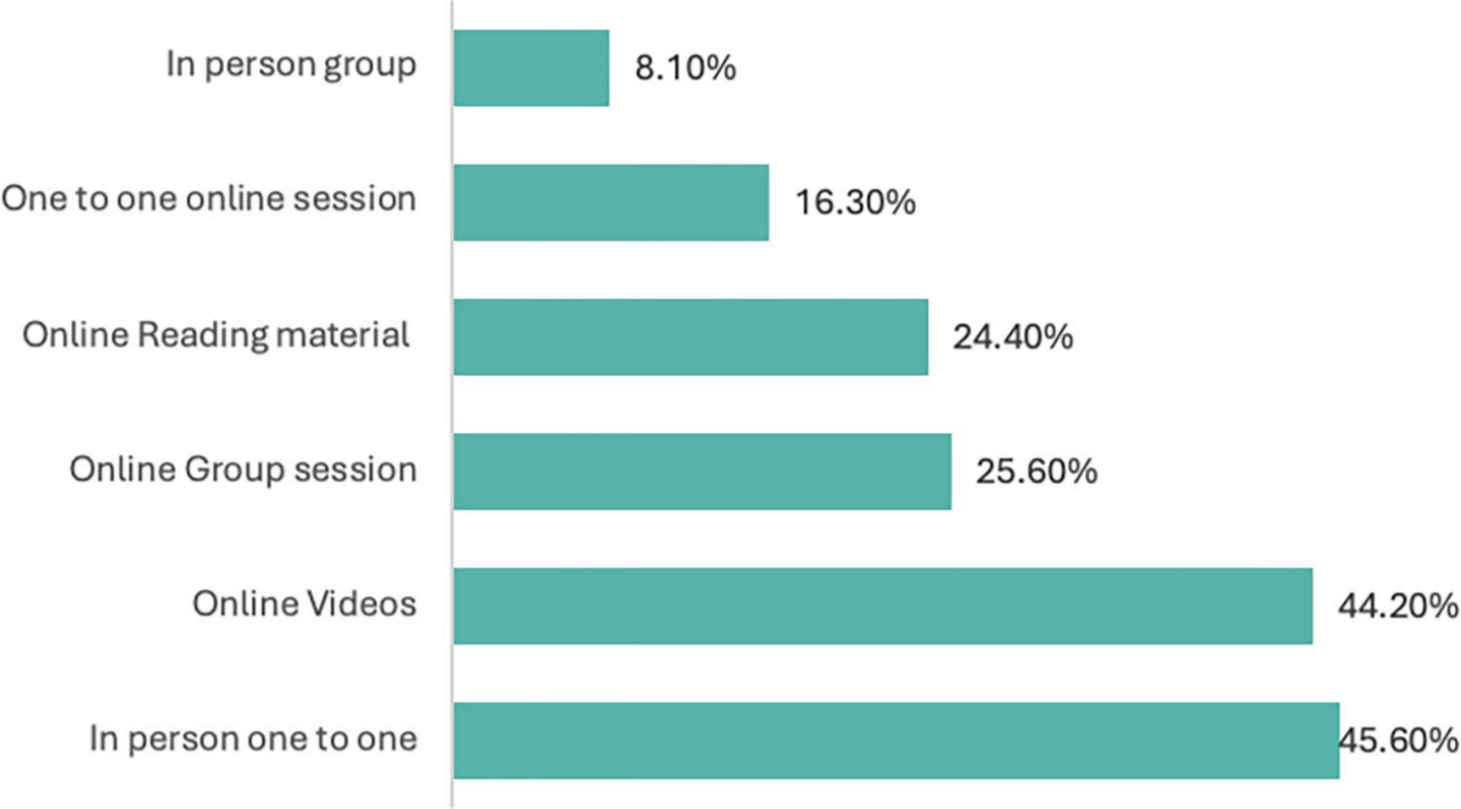
Preferred mode of delivery for exercise and PA education intervention.

Fig 3 depicts all other modes of delivery chosen.

## Discussion

To our knowledge, this is the first study to investigate the perspectives of individuals living with PD on exercise and PA promotion interventions, as well as their knowledge levels and priorities regarding exercise education. By assessing PwP’s views, knowledge levels, and preferences, this research fills a crucial gap in the literature, providing valuable insights for the development of tailored interventions. Responses from PwP highlight the lack of apparent provision of education and exercise intervention from the National Health Service, and a potentially large unmet need. PwP in this study report that they often do not understand the exercise and PA information they receive and underscore the lack of signposting to local support or exercise groups. Among participants, general knowledge about the importance and benefits of exercise for both motor and non-motor symptoms is moderately high, yet awareness of the impact of PD on posture, muscle strength and falls is low. The survey results also underscore that PwP believe an education intervention should be provided for people with a new diagnosis of PD. They prioritise content that focuses on translating the latest research findings into practice as well as content that is relevant to their symptoms and abilities, delivered in person or online.

The lack of access to information and services in the UK around the time of diagnosis for PwP has been previously identified in the literature [27, 28]. A recent study highlighted that exercise and PA interventions are delivered by physiotherapist on average 3 years post diagnosis [29]. The current survey highlights the extent of this, as well as the potential health inequalities in accessing care in this population and personal economic burden of PwP [30], with 56% of participants reporting they had paid privately to access education and exercise classes. Our findings also show that many PwP may not have the knowledge or skills to change their exercise or PA behaviours on their own, echoing previously reported mismatch between perceived and actual knowledge on PD [31]. For example, participants reported being aware of the overall benefits of exercise and PA, however failed to identify the differences between exercise and PA with 90% identifying activities such as walking the dog as exercise. These misconceptions may have practical implications when clinicians advise or deliver interventions around these concepts in clinical practice.

While 78% of participants reported to have a good understanding of the role of exercise in the management of PD, 90% of participants somewhat to strongly agreed they would participate in education interventions that focus on the specific elements of why and how to exercise with PD, highlighting the need for interventions featuring tailored and relevant PA content, accompanied by practical recommendations for implementation [32].

Lack of understanding of the type, intensity or frequency of activity, and a lack of available resources such as Parkinson’s specific classes, are known barriers to behaviour change in PD [28, 33]. This indicates that the role of the clinician may need to go beyond stating the importance of exercise and PA and focus on improving the capability and self-efficacy of PwP to increase their activity levels. Although well positioned to promote non-pharmacological interventions for the management of PD symptoms, healthcare professionals report lack of time and confidence to be some of the barriers in delivering specific and tailored interventions that promote PA in population [29]. This was also highlighted in the recent Parkinson’s UK audit. The audit indicated that 1 in 5 consultants had not taken a PD specific continuous professional development course and identified that improvements in educating the workforce are required [34].

This study explored the knowledge and understanding of the role of exercise in the management of PD symptoms to identify the extent to which knowledge of the benefits of exercise can impact self-report exercise participation levels. Behavioural models such as the Capability Opportunity and Motivation (COM-B) [35] discuss the need to improve capability, i.e. knowledge on the role of exercise and PA as well as opportunity and motivation, to enable first participation and then maintenance of lifelong healthy behaviours [20, 36]. However, most interventions informed by these theories do not tend to explore the impact that physical literacy has in driving exercise and PA participation in PD [17]. As a secondary objective, this relationship was explored in this study with no significant relationship detected between the level of knowledge and self-reported minutes of MVPA. This may be due to a number of limitations such as potential social-desirability bias, self-selection bias, reliability or validity of the KEPA questionnaire [37]. Although previous studies have explored different theoretical models to identify potential determinants in exercise behaviour both in older healthy adults [38] and in people with PD [24], future interventions aiming to promote PA and exercise in PwP should explore the role of physical literacy as a likely determinant of activity behaviour if theoretical behaviour models such as COM-B are used.

### Strengths and limitations

The results from this cross-sectional study provide valuable insights but need to be interpreted with caution, especially by not attempting to establish causality due to potential confounding and unmeasured factors but also when assessing the generalisability of the results to the wider Parkinson’s population. Although there was a good representation of both male and female respondents, the majority were highly educated (i.e. at university level of above) and were of white background. A higher proportion of participants with higher level of education has been reported in several PD studies [31, 39, 40]. This may be attributed to the potential correlation between higher level of education and the risk of developing PD [41] and/or to the underrepresentation of individuals from lower socioeconomic and ethnic minority backgrounds, both in Parkinson’s-specific research [42] and research overall [43]. Despite best efforts, responses from ethnic minorities were limited. Also, the survey was conducted online therefore self-selection bias needs to be considered when interpreting findings.

Most participants self-reported to engage in 200 or more minutes of MVPA per week. This may be due to a social desirability bias, i.e. an overestimation of PA when it is subjectively measured, which is a common occurrence in the literature [44]. It may also be an indication of participation bias in the survey which has a focus on exercise and PA, and therefore, might attract people who have an interest in, and regularly exercise. Furthermore, the heterogeneity of PD in terms of symptom presentation and progress make it difficult to achieve consensus in education topic priorities as these vary depending on confounders such as socioeconomic factors, baseline physical activity levels, comorbidities etc. However, the study did not seek consensus on the subject but aimed to explore and describe the physical literacy priorities of PwP, providing practice relevant insight to improve care for newly diagnosed PwP. Additional work is needed to explore these aims further, and importantly, to ensure that interventions are designed based on the needs of the PwP and content is not merely generated on clinical intuition and knowledge of existing literature.

Notwithstanding the limitations, the survey was completed by more than 400 PwP and had a 94% completion rate, which mitigates some of the limitations and moreover, provides considerable insight on a subject that has not been previously studied in this population.

## Conclusions and implications for practice

The study has highlighted the need for exercise and PA interventions around the time of diagnosis for PwP in the UK. Previous studies have discussed why it is important for PwP to receive content that is specific and tailored to their needs [32, 45]. Future interventions promoting exercise and PA should include content that aims to improve physical literacy with tailored content around how PD may impact muscle strength, balance, and posture and how exercise and PA can mitigate motor as well as non-motor symptoms. Participants prioritised seven important topics to cover when promoting exercise and PA and have identified online videos and in person sessions as an acceptable mode of delivery. These findings provide a firm evidence-base starting point to guide the development of exercise and PA promotion intervention to address current knowledge gaps, and the needs and preferences of people living with PD.

## Supporting information

S1 Table Survey questions and objectives. (PDF)

S1 Fig Education/information provision on exercise and PA grouped by years living with PD. (TIF)

S2 Fig Perceptions and beliefs around the role of exercise and PA in Parkinson’s disease. (TIF)
