# Supplementary material for: Exploring the experiences, priorities and preferences of people living with Parkinson’s on exercise and physical activity promotion in the UK
Source: PLoS One. 2024 Jun 12;19(6):e0304223. doi: 10.1371/journal.pone.0304223 (PMC11168645; doi:10.1371/journal.pone.0304223)
Supplement: S1 Table — (PDF) [file pone.0304223.s001.pdf]

S1: Survey questions and objectives

Objective	Survey questions	Answer type
**A. Demographics**	**What is your age?**<br>**What is your gender?**<br>**How would you describe your ethnic group?**<br>**What is the highest education qualification you have achieved?**<br>**How long ago where you diagnosed with Parkinson's?**<br>**Have you been diagnosed with any other physical or mental health condition?**<br><br>**Which of the following statements describe best how Parkinson's affects your daily activities?**<br>**-** No difference in my day-to-day activities.<br>- Some difficulty in day-to-day activities. I have noticed although I am independent, I am slower in my movement.<br>- Moderate levels of difficulty with day-to-day activities. I am mostly independent but require assistance for demanding activities.<br>- High levels of difficulty with day-to-day activities. I require assistance with most activities of daily living.<br>- Extreme difficulty with daily activities. I am fully dependent for all daily activities.<br>- Other, please specify.<br><br>**In the last week, how many minutes,** did you spend doing **moderate to vigorous** physical activities? (activities that cause increase in breathing, for example brisk walking, pushing a lawnmower or cycling uphill)<br><br>**0-200**	Free text or multiple choice (one answer possible)

S1: Survey questions and objectives

B. **To explore the practice around education/information provision for PwP in the UK**	**Around the time of diagnosis:**<br>1. I received a satisfactory amount of information about the benefits of exercise in Parkinson's.<br>2. I was given a satisfactory amount of information about available classes and activity groups near me.<br>3. I was given an exercise programme specific to my needs and symptoms.<br>4. I understood the exercise information I was given.<br>5. I knew which exercises to complete in order to manage my symptoms.<br>6. I knew what outcomes to expect from the exercises I was given.<br>7. I was confident I had a good level of knowledge about the role of exercise in Parkinson's.<br>8. I wanted to know more about the role of exercise and physical activity in Parkinson's.<br>**If you want to find out information about exercise and PA where do you look?**	Strongly Disagree<br>Somewhat disagree<br>Neither agree or disagree<br>Somewhat agree<br>Strongly agree
	1. When first diagnosed, people with Parkinson's are 30% less active than the general population of same age.<br>2. Regular aerobic exercise may improve abilities such as thinking and reasoning.<br>3. Reduced levels of physical activity are associated with a reduction in social interactions over time in people with Parkinson's.	Agree<br>Not Sure<br>Disagree

**C. To identify the gaps in the knowledge level of PwP around exercise and PA.**	4. Loss of arm swing is connected to the shuffling walking type that is common in Parkinson's.<br><br>5. Half of people with Parkinson's can experience depression and anxiety.<br><br>6. Muscle strength is not reduced in the early stages of Parkinson's.<br><br>7. Moderate to high intensity exercise are not recommended as they can negatively impact Parkinson's.<br><br>8. Walking the dog and gardening are a form of exercise.<br><br>9. People with Parkinson's fall as much as the general population of the same age.	
**D. To identify the needs and preferences of PwP on exercise and PA education provision around the time of diagnosis.**	**At the present moment, how much do you agree or disagree with the following statements:**<br><br>1.I am knowledgeable about the role of exercise in Parkinson's.<br><br>2. As Parkinson's progresses, the type of information around the exercises I need, change.<br><br>3. Knowing which specific exercises to do is important to me.<br><br>4. Parkinson's is a progressive disorder and that is why exercise, although useful, cannot slow down the course of Parkinson's.<br><br>5. Providing too much information about Parkinson's can be unhelpful.<br><br>6. People with Parkinson's can have a good quality of life.<br><br>7. Exercise is only effective if is started at the early stages of Parkinson's.<br><br>8. I feel the same whether I exercise or not.	Strongly Disagree<br>Somewhat disagree<br>Neither agree or disagree<br>Somewhat agree<br>Strongly agree

	9. If an education programme focusing on why and how to exercise was available, I would participate.	
**D. To identify the needs and preferences of PwP on exercise and PA education provision around the time of diagnosis.**	**How do you prefer information or education programmes about exercise and physical activity to be delivered to you?**<br>○ In person one to one session with clinician (please state which clinician)<br>○ Online session one to one with clinician (please state which clinician)<br>○ Online videos<br>○ Online reading materials<br>○ Online group sessions<br>○ Other (please specify<br><br>**Which of the following topics would you include in an education programme that aims to provide information about exercise and physical activity for a person newly diagnosed with Parkinson's?**<br>○ Current evidence on why exercise is important in Parkinson's.<br>○ How exercise impacts on motor symptoms (stiffness, posture) and non-motor symptoms (sleep, depression).<br>○ The best exercises for my symptoms.<br>○ What is classed as exercise and what as physical activity?<br>○ Evidence of the role of other activities such as dancing, tai-chi and Nordic walking on Parkinson's.<br>○ Setting meaningful goals for me.<br>○ How to progress my exercise programme.<br>○ What I can do, if I am not interested in exercise.	Multiple choice (up to 2 options)<br><br>Multiple choice (up to 7 options)

	○ Falls risks in Parkinson's and how to manage them.<br>○ When I would I see the benefits of exercising.<br>○ Exploring common barriers to exercise and how to overcome these.<br>○ Information about support groups and activities near me.<br>○ Pain, stiffness, balance: what is normal part of ageing, and what is Parkinson's.<br>○ How to work collaboratively with the healthcare professionals to help achieve my goals.<br>○ Ways to motivate myself to exercise more.<br>○ Other (please specify)	

.
