# Supplementary figures and images for: Exploring the experiences, priorities and preferences of people living with Parkinson’s on exercise and physical activity promotion in the UK
Source: PLoS One. 2024 Jun 12;19(6):e0304223. doi: 10.1371/journal.pone.0304223 (PMC11168645; doi:10.1371/journal.pone.0304223)

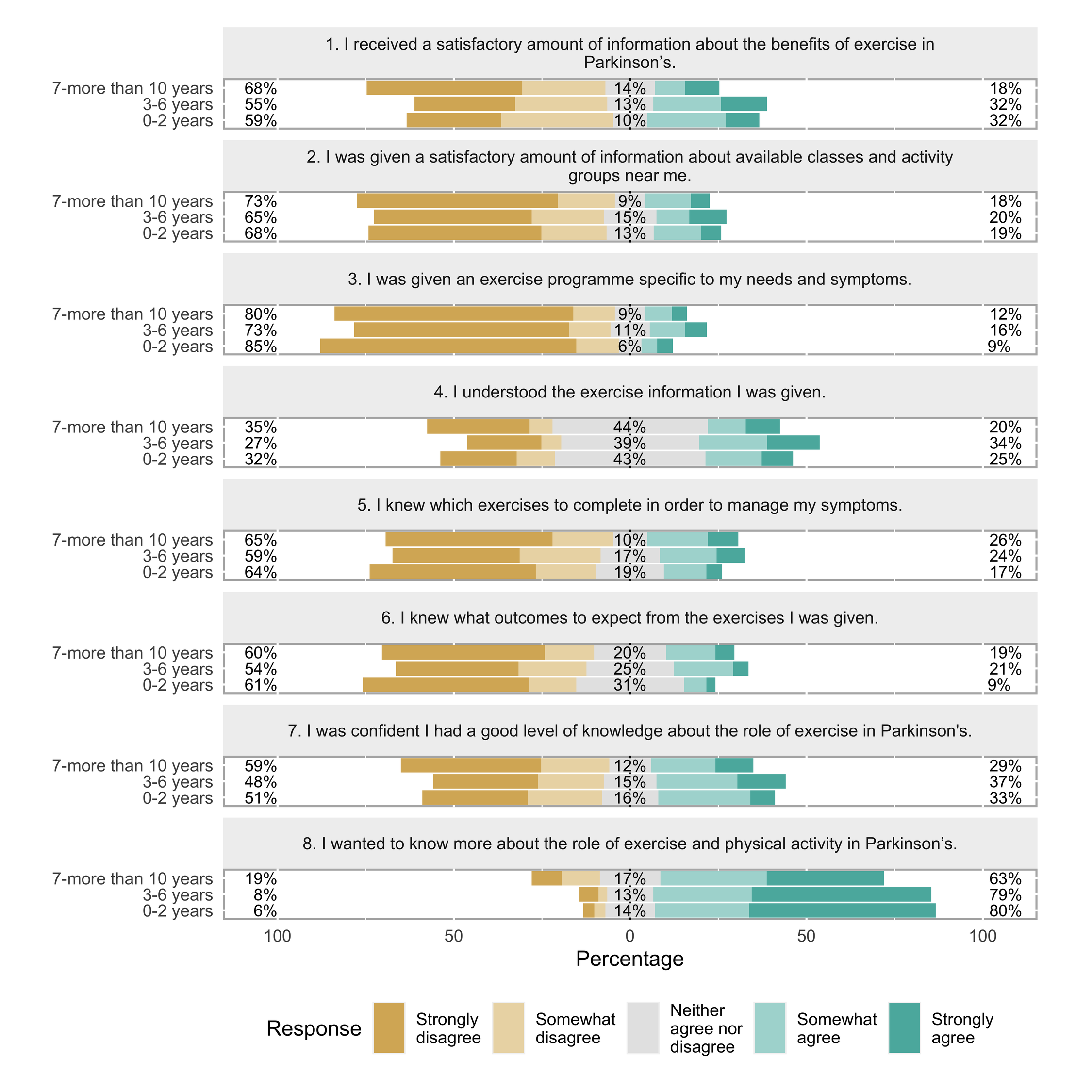

Supplement: S1 Fig — (TIF) [file pone.0304223.s002.tif]

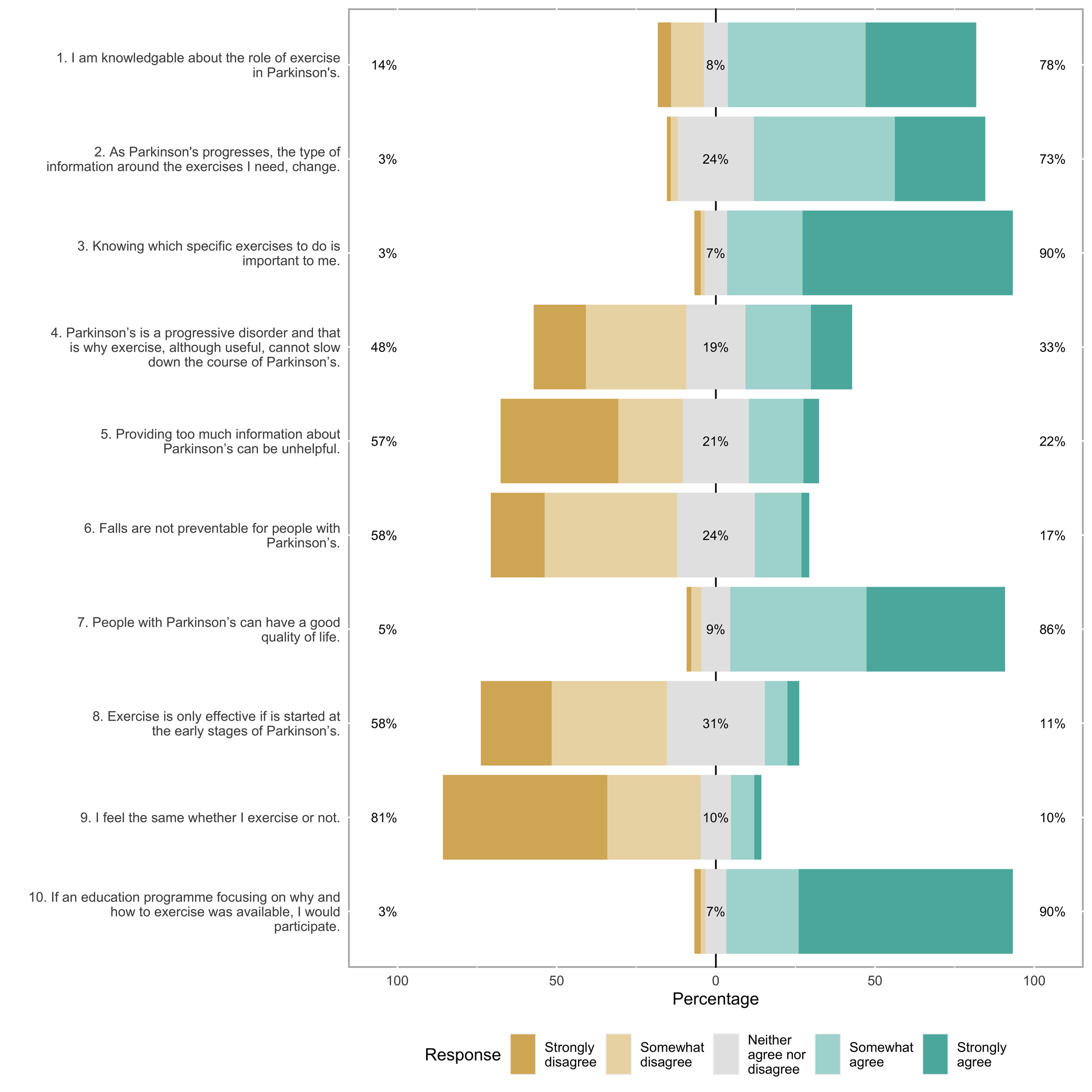

Supplement: S2 Fig — (TIF) [file pone.0304223.s003.tif]
